# Editorials

**Published:** 1890-04

**Authors:** 


### HOMŒOPATHIC PHYSICIAN,

A MONTHLY JOURNAL OF

HOMŒOPATHIC MATERIA MEDICA AND CLINICAL MEDICINE.

If our school ever give up the strict inductive method of Hahnemann, we are lost, and deserve only to be mentioned as a caricature in the history of medicine."—CONSTANTINE HERING.

Vol. X. APRIL, 1890. No. 4.

### EDITORIALS.

THE INTERNATIONAL CONGRESS.—We give this month an extended summary of the proceedings of the Homœopathic Congress held at Paris last August. These discussions were so interesting that, notwithstanding their obvious departures from the standard of the strict Homœopathy of Hahnemann, we believe they will be acceptable to our readers, all of whom are too well grounded in the knowledge of the genuine principle to be misled into practicing the errors several of these gentlemen teach. The discussions were carried on in French, but, as we were unable to get a sufficiently detailed report in that language in time, we had recourse to a translation that had been made from French into Spanish and published in a medical journal called *El Consultor* at Barcelona. These we have had again translated into English for our own readers.

This report is instructive as exhibiting vividly the fantastic heresies that have grown like parasites upon Homœopathy. It shows how difficult it is for a comprehension of the plain principles of Hahnemann's system to penetrate the minds of most men. It gives us some idea of who among Europe's distinguished physicians are and who are not strict followers of the inductive method of Hahnemann. Here and there through the discussions we may discover true Hahnemannian sentiments, and toward the end a sharp rebuke is administered to one of the worst of these heresies, which is greatly to the credit of the Congress.

Therefore, while we are unable to indorse any of the departures from the true standard that were defended in the meetings, and disappointed that there was not a unanimous sentiment at the last for the cause of Hahnemannian Homœopathy, we nevertheless think that from a perusal of the report some important precepts may be gathered, many instructive lessons may be learned.

On the whole, the proceedings are a striking pen-picture of the progress of medicine in the new school.

We shall be glad to receive comments for publication from any of our subscribers upon any question raised in these notes of the discussions. W. M. J.

---

The Value of Trivial Symptoms is so great that every Hahnemannian can attest that brilliant cures have resulted in difficult cases from paying attention to, and finding a remedy in whose pathogensis such symptoms are alone found. It is only the true disciple of Hahnemann who is able to estimate these symptoms at their real worth. But there is frequently a difficulty in eliciting such symptoms. The patient, thinking them unimportant, fails to mention them, and the physician, hesitating about leading questions, will fail to know of their presence. A perplexing case will offer; all the elements of the case are listened to and noted. The totality of the symptoms and of each individual symptom are related, and yet the trouble will be to find the remedy most similar to the case. Then, as if thinking it of no worth, the patient, sometimes in doubt about the necessity of mentioning such a trifle—as it appears to him—will give a clue to the only fit remedy for his entire affection by stating that some slight symptom is present.

The true physician will then know if it be of worth, and, finding it so his labor will be rewarded when the remedy to which this symptom belongs is found.

Not only this. He will find under that same remedy all the symptoms peculiar to the case.

By noting just these apparently trifling symptoms Hahnemann proved himself a genius. And any one can put this proof to the test by imitating his example.

And yet for this important work Hahnemann received only ridicule, and because of the abuse, and hatred, and ignominy which was showered upon him he left his native land, to find a home and friends in a foreign country.

That he was far ahead of his contemporaries, his keen observation of facts which physiology since then has proved to be correct will bear witness.

We need no stronger proof of this than some of the observations he made, while proving remedies, on the value of the various symptoms generated by them.

Here we may see an acuity of vision, and a profundity of thought far beyond that of any of his day. Indeed, even with the physiological knowledge of the present day, only his followers are qualified to accept at their real merit physiological facts.

We can find no better illustration of this keen vision and deep thought than by turning to the provings of Arsenicum, Ignatia, and Phosphorus.

Merely mentioning mental symptoms, of the supreme value of which every homœopathician knows, let us turn to the symptoms of hearing.

Under Arsenicum we find : Hardness of hearing, cannot hear the human voice ; but other sounds can be heard.

Under Ignatia : Hard hearing except for speech.

Under Phosphorus : Hardness of hearing, especially for the human voice.

It is such symptoms as these that have been laughed at, even by some who call themselves homœopathists. They say, "if they do exist, which we doubt, they can be of no importance." But we know they are of value, and their significance can be shown by turning to any modern work on physiology. And we may there see how the genius of Hahnemann is confirmed by the best physiologists of this day.

Within a very recent period more has been known of the function of hearing through the work of an ingenious Italian, Corti, and by the labors of Helmholtz. Corti discovered and described that part of the hearing apparatus known as the organ of Corti, and Helmholtz, by his toil, has shown the functions of this organ, and what an essential share it has in the sense of elaborating the pitch of various sounds.

This organ lies in the centre of the floor of canalis cochlearis, which is formed by the membrana basilaris, and it receives the terminal filaments of the nervus cochlearis. In the centre of the organ of Corti are the rods of Corti, which are firm elongated bodies whose bases rest upon the membrana basilaris.

The radiating fibres composing the membrana basilaris are compared to the strings of a harp or piano, and Helmholtz advances the theory that each fibre is attuned to vibrate in unison with a note of a definite pitch. It communicates its vibrations to the corresponding cells in the organ of Corti. It is said that the function of the rods of Corti is probably to take up the vibrations from the labyrinthine fluid and the fibres of the membrana basilaris, and transfer them as a nerve irritation to the terminal filaments of the auditory nerve. Varying in length and span, their number is sufficient to allow four hundred or more rods to each octave within the musical scale. (See article Hearing, *Reference Hand-book of the Medical Sciences.*)

Of the construction and function of these organs nothing was known in Hahnemann's day; but that sagacious observer was able to detect even the slightest alteration in such a complex function.

We might continue this subject indefinitely, and treat of all the known functions to witness the acumen of the man we follow, but we think we have shown enough to bear out the assertion we made at p. 56, February number, that physiological facts go to confirm all of Hahnemann's observations. G. H. C.

